# Brain access of incretins and incretin receptor agonists to their central targets relevant for appetite suppression and weight loss

**DOI:** 10.1152/ajpendo.00250.2023

**Published:** 2024-02-21

**Authors:** Sophie Buller, Clemence Blouet

**Affiliations:** Medical Research Council (MRC) Metabolic Diseases Unit, Institute of Metabolic Science, University of Cambridge, Cambridge, United Kingdom

**Keywords:** brain access, incretins, obesity, weight loss

## Abstract

New incretin-based pharmacotherapies provide efficient and safe therapeutic options to curb appetite and produce weight loss in patients with obesity. Delivered systemically, these molecules produce pleiotropic metabolic benefits, but the target sites mediating their weight-suppressive action are located within the brain. Recent research has increased our understanding of the neural circuits and behavioral mechanisms involved in the anorectic and metabolic consequences of glucagon-like peptide 1 (GLP-1)-based weight loss strategies, yet little is known about how these drugs access their functional targets in the brain to produce sustained weight loss. The majority of brain cells expressing incretin receptors are located behind the blood–brain barrier, shielded from the circulation and fluctuations in the availability of peripheral signals, which is a major challenge for the development of CNS-targeted therapeutic peptides. GLP-1 receptor (GLP-1R) agonists with increased half-life and enhanced therapeutic benefit do not cross the blood–brain barrier, yet they manage to access discrete brain sites relevant to the regulation of energy homeostasis. In this review, we give a brief overview of the different routes for peptide hormones to access the brain. We then examine the evidence informing the routes employed by incretins and incretin receptor agonists to access brain targets relevant for their appetite and weight-suppressive actions. We highlight existing controversies and suggest future directions to further establish the functionally relevant access routes for GLP-1-based weight loss compounds, which might guide the development and selection of the future generation of incretin receptor polypharmacologies.

## Graphical abstract

**Figure:**
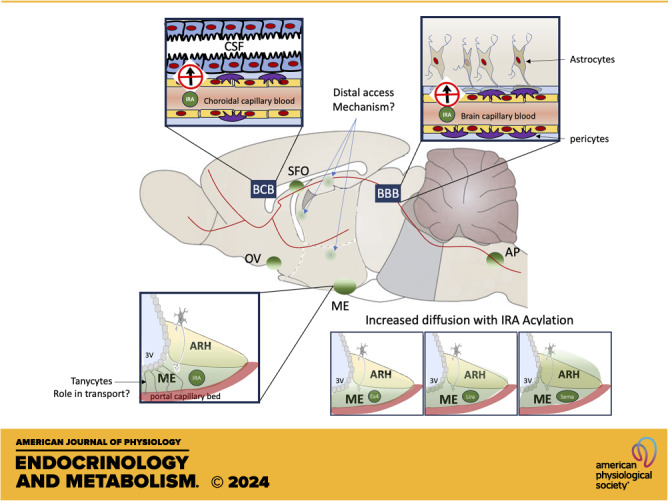

## BRAIN ACCESS OF PEPTIDE HORMONES

Studies characterizing brain entry of peptide hormones form a good basis to think about how incretin-based pharmacotherapies access their brain targets, how access might be regulated under physiological and pathological conditions, and the pros and cons of various tools to visualize access with high neuroanatomical resolution and/or quantify brain entry of these molecules. Here we first briefly review the literature characterizing the different access routes employed by peptide hormones to access their brain targets, with a focus on hormones relevant to energy homeostasis such as leptin.

### The Blood–Brain Barrier

Drug access to the brain is highly restricted by brain barriers gating the entry of circulating molecules and critical for the brain’s homeostasis. The blood–brain barrier (BBB) is a specialized multicellular structure that forms a regulatory interface between capillaries and the brain parenchyma. Capillary endothelial cells of the BBB are linked by a network of strongly embedded tight junction proteins that seal paracellular gaps and restrict the transfer of most substances from the bloodstream into the brain (1). Brain endothelial cells maintain close interactions with surrounding astrocytes, pericytes, and perivascular macrophages, together forming the neurovascular unit (1). BBB passage of circulating biomolecules is achieved via paracellular or transcellular mechanisms. The paracellular route relies on passive diffusion across the BBB; however, successful transport via this pathway is limited to small (approximately <500 Da) lipophilic molecules (2). Since most drugs and peptide hormones are too large to passively diffuse across the BBB, they instead rely on alternative access mechanisms through barrier cells that are dependent upon the expression of specific transporters on BBB endothelial cells and transcytosis (1).

Changes in circulating levels of peptide hormones can be detected throughout the brain following receptor-mediated access across the BBB. For the hormone leptin, this can be unequivocally concluded from the fact that following peripheral leptin administration, p-STAT3, a specific marker of active leptin signaling, is detected in leptin receptor-expressing cells distributed in multiple areas of the brain located behind a strict BBB (3). Direct evidence for active receptor-mediated transport systems has been obtained for several metabolic hormones including leptin and insulin using radioactively labeled peptides and multiple-time regression analysis, allowing a detailed and highly specific characterization of key transport features across the BBB brain (4). Through this route, transport to the brain is slow and saturable. As an example, leptin levels in the parenchyma reach 5% of circulating concentrations in lean mice following a pharmacological bolus (4).

Intriguingly, recent advances in the understanding of BBB function support a significant degree of vascular heterogeneity in different brain regions, and a significant role for all cells of the neurovascular unit in barrier functions. CNS pericyte loss, for example, has been shown to correlate with BBB permeability (5). Pericytes and other cells of the neurovascular unit express receptors for metabolic hormones, and recent evidence highlights a role for pericyte leptin signaling in the regulation of paracellular transport across the BBB (6), suggesting that under different metabolic conditions, BBB permeability to circulating metabolic signals might vary.

### The Blood–Cerebrospinal Fluid Barrier

Circulating substances also enter the brain through the choroid plexus, a network of blood vessels and modified ependymal cells distributed in the ventricles that produce cerebrospinal fluid (CSF) (7). The Choroid Plexus capillaries, which are fenestrated to allow free diffusion of small blood-borne molecules, and ependymal cells, joined together by tight junctions, form the blood–CSF barrier (BCB). The movement of substances from the blood into the CSF is in many ways analogous to that from the blood into the brain, with many of the same transporters present in both tissues. For example, the short form of the leptin receptor, functionally acting as a transporter, is expressed by choroid plexus ependymal cells (8) and provides a route of access to the brain for leptin (9). Once in the CSF, leptin binds to leptin receptors expressed in ependymal cells and crosses through the ependymal barrier into the brain parenchyma (10).

Circumventricular organs (CVOs) are discrete brain structures bordering the third and fourth ventricles devoid of a functional BBB. They provide additional sites of blood-CSF exchanges regulated by ependymal cells, which form a barrier along ventricular walls (11). CVOs are designed to facilitate neurohumoral communications between the brain and periphery and offer an alternative route for peptides and hormones to enter the brain. Peripherally injected drugs freely diffuse into and rapidly accumulate in CVOs. This can be nicely visualized with high neuroanatomical resolution by whole brain clearing combined with light sheet microscopy following the peripheral administration of fluorescently labeled compounds (12). Thus, CVOs have exclusive access to unbuffered concentrations of circulating factors including nutritional and hormonal signals and exogenously delivered compounds. Here, we will discuss how metabolic signals access the brain with a particular focus on the median eminence (ME) in the hypothalamus and the area postrema (AP) in the hindbrain, two CVOs of most relevance in the context of energy balance regulation given their proximity to interoceptive regions critical for metabolic homeostasis.

### CVOs, a Privileged Access Route for Drugs and Metabolic Hormones

From the CVOs, brain targets can be exposed to circulating hormones and drugs through several mechanisms in addition to the BCB route. Front row cells residing in CVOs benefit from direct, unbuffered access to the bloodstream, exposing them to all compounds present. The AP is densely populated with neurons relevant to energy homeostasis that project locally to engage appetite-regulating circuits in response to changes in concentrations of metabolic signals (13). In contrast, the ME is highly enriched in nonneuronal cells, which represent over 90% of ME cells (14). They include oligodendrocyte lineage cells, astrocytes, and pericytes, all of which have been shown to contribute to metabolic sensing and energy homeostasis (6, 15–18). Recent work characterizing the unique characteristics of ME oligodendrocytes lineage cells, which are transcriptionally homogeneous but functionally distinct from their ARH counterparts in terms of rates of proliferation, differentiation and maturation, highlights the functional consequences of the enriched CVO environment on glial biology and its consequences on energy homeostasis and metabolic sensing (14, 17, 18). Additional work is, however, needed to better characterize the role of CVO’s nonneuronal cells in the regulation of brain access and sensing of metabolic signals.

Large populations of metabolic sensing neurons located in the ARH and NTS are separated from their respective CVOs by diffusion barriers, which block the passage of molecules between the ME and ARC or AP and NTS, respectively (8). This can be clearly visualized following the injection of blood–brain barrier impermeant tracers such as Evans Blue (11) or dextrans of various molecular weights. These studies indicate that molecules 3 kDa or larger do not freely diffuse from the ME to the ARH to adjacent parenchymal regions (19). Tanycytes present at CVO-parenchymal barriers have been proposed to form these diffusion barriers (20, 21). However, the ME-ARH barrier is maintained in mice with inducible ablation of mediobasal hypothalamic tanycytes, suggesting that alternative cell types form the ME-ARH barriers (22), or at least are engaged as a compensatory mechanism to maintain the barrier following tanycyte ablation. Additional models would be needed to rule out the role of tanycytes in the ME-ARH barrier of neuroanatomically intact animals. Thus, the molecular and cellular architecture and exact location of CVO-parenchymal barriers remain unresolved. Nevertheless, circulating molecules can diffuse to at least some territory within the ventromedial ARH or NTS, and the diffusion properties of the barriers bordering these regions are plastic and regulated by nutritional and circadian signals (20, 23–25). In addition, there is clear evidence that a large proportion of ARH neurons can directly access the CVO environment (26, 27). For example, half of leptin receptor-expressing neurons of the ARH are labeled with BBB-impermeant retrograde virus Fluorogold, indicating that they are directly exposed to circulating molecules (27). Some of this labeling likely results from tracer uptake by dendritic or axonal projections to the ME, since neurons located in lateral portions of the ARH are also labeled (27). In fact, diverse hypothalamic neuronal populations extend dendrites to the ME, or contact the ME through axonal terminations. This is the case for PVH neuroendocrine neurons, for example, which are also densely labeled following peripheral fluorogold administration (27). Thus, multiple hypothalamic populations are strategically positioned to be protected from potential circulating pathogens, glucotoxic, and lipitoxic stresses, and yet close enough to access relevant information in the blood.

Intriguingly, ME tanycytes lining the ventral wall of the third ventricle, which form the ME BCB, have been proposed to provide a third privileged access route for circulating signals to enter the hypothalamus via transcytosis through the ventricular space and potentially deliver high concentrations of leptin to nearby parenchymal areas through local CSF (28). However, although good evidence indicates that ME tanycytes take up circulating hormones such as leptin (29), the expression of the leptin receptor and leptin-induced pSTAT3 in tanycytes is controversial (28, 30). Although this topic is outside the scope of this review, these data highlight some of the technical challenges associated with the functional characterization of lowly-expressed receptors in tanycytes. One of the major limitations is the lack of models allowing efficient and specific knockout of relevant transport mechanisms in ME tanycytes. Ventricular Tat-Cre injections, a cell-permeable recombinase, or expression of Cre recombinase under the *Dio2* promoter, are efficient strategies to target ME tanycytes and produce relevant functional consequences in the hypothalamus (29). However, both strategies produce recombination across ependymal cells and in the choroid plexus, and might widely alter transporter expression across brain barriers (31, 32). Inducible Cre expression under the *Rax* promoter might provide increased tanycytic specificity but needs more extensive characterization (30, 33). Given these controversies, future studies should employ histological techniques with higher neuroanatomical resolution, more systematically examine the brain-wide consequences of loss-of-function models, provide extensive validation of various receptor expression and intracellular signaling detection strategies using positive and negative controls, and examine the role of ME tanycytes in brain access to sites more distal from the ME and involved in a wider range of functional consequences.

Dose–response and time-course studies have been critical to distinguish the conditions under which access through the various entry points (CVOs vs. BBB vs. BSB) is most relevant. Studies examining leptin transport under different conditions are particularly insightful and support the conclusion that access of leptin through the ME to the ARH is designed to rapidly respond to a lower range of physiological leptin concentrations. ARH neurons exhibit active leptin signaling even in the absence of exogenous leptin administration in lean mice, while other brain sites do not (27). On the other hand, leptin signaling in the ARH saturates following an intraperitoneal dose of 50 ug/kg, while showing a linear dose-dependent increase until at least 1 mg/kg in other hypothalamic and extrahypothalamic leptin-sensitive sites (34). Leptin resistance is another specificity of ARH leptin-sensing neurons and does not develop in other hypothalamic and extrahypothalamic sites, even in mice maintained on a high-fat diet for 16 wk (35). Thus, the ARH is not designed to respond to increases in peripheral leptin levels and better responds to changes in leptin within the lower range of physiological concentrations (26). To date, there is no indication that central resistance to incretins exists, yet these studies illustrate some unique features of the ARH, which make this site most relevant for rapid and accurate sensing of levels of circulating signals within the normal physiological range.

### Disruptions of Brain Barrier in Obesity and Metabolic Disorders

Several pathological conditions, such as obesity, inflammation, and metabolic disorders, can disrupt brain barriers and alter the regulation of peptide hormone access to their target sites. For example, the ratio of CSF leptin levels to serum leptin levels is four times greater in lean than individuals with obesity, indicating saturation of the BBB/BCB routes in these conditions (36–38). In addition, altered diffusion through the ME-ARH barriers contributes at least in part to diet-induced leptin resistance in the ARH, since ARH neurons maintain some responsiveness to centrally administered leptin but fail to produce the expected rapid increase in leptin signaling following peripheral leptin administration (28, 39). Intriguingly, a pharmacological bolus of fluorescently labeled leptin equally accumulates in the CVOs and choroid plexus of lean versus obese mice, against the idea that impaired brain leptin access in obesity contributes to leptin resistance (12). Additional investigations across a range of leptin doses and at various timepoints after the leptin bolus might provide some insights into the sensitivity of this technique to reliably quantify changes in brain leptin transport.

## BRAIN ACCESS OF GIP, GLP-1, AND ASSOCIATED PHARMACOTHERAPIES

### Is Brain Access of Gut-Derived GIP, GLP-1, and Incretin-Based Therapies Relevant for Their Central Action?

The rapid degradation of peripheral incretins by circulating dipeptidyl Peptidase IV (DDP-IV) is often used as evidence against a potential contribution of brain incretin receptor signaling in the central consequences of gut-derived GLP-1 and glucose-dependent insulinotropic peptide (GIP) in lean conditions. Primarily a postprandial signal released in response to nutrient sensing, circulating GIP also builds up in response to chronic high-fat feeding, creating a physiological situation under which increases in circulating GIP levels almost certainly reach the brain (40). CNS deletion of the GIP receptor (*Gipr*) phenocopies the feeding and metabolic profile of global germline knockout (41), providing further support for the relevance brain GIPR signaling in the feeding and metabolic consequences of endogenous GIP release.

This question remains unsettled for GLP-1 [detailed in excellent reviews (42, 43)] given that sustained elevation in gut GLP-1 production has not been reported outside the artificial context of gastric bypass surgery (44). It is generally admitted that in the postprandial context in healthy animals, gut-derived GLP-1 signals to the brain by acting on vagal afferents given that circulating GLP-1 is degraded by DPP IV within minutes (45). Thus, direct brain sensing of gut-derived GLP-1 seems unlikely (46). In addition, brain or neuronal knockout of *Glp1r* does not produce energy imbalance (47–49); however, developmental compensations likely limit the usefulness of these models in understanding the role of brain GLP-1R signaling in the central action of gut-derived GLP-1. In fact, virally mediated knockdown of NTS *Glp-1r* in adult rats increases spontaneous weight gain (50). Nevertheless, whether discrete brain areas (such as the CVOs) might be exposed to the transient elevation in circulating GLP-1 remains unclear. As detailed above, the literature on ARH leptin sensing makes a strong case supporting a role for the ME-ARH region in the rapid detection of physiological changes in circulating levels of metabolic hormones. The fact that the ME-ARH is exposed to changes in circulating GLP-1R agonists within 1 min post intravenous dosing (51) suggests that local neurons might be exposed to postprandial surges in endogenous gut-derived GLP-1 before it is completely degraded. Strikingly, the ME and AP display some of the highest density of GLP-1R immunoreactivity in the brain (52), but receive little inputs from brain GLP-1-producing neurons (53), begging the question of the role of these receptors in normal physiology. Thus, additional investigations, ideally using site-specific inducible loss-of-function models in adults, are needed to fully rule out a contribution of the direct brain sensing of gut-derived GLP-1 in its physiological action.

In contrast, there is clear evidence that brain signaling is required for the feeding and metabolic actions of incretin-based weight loss agents (41, 54). Discussing the routes of brain access relevant for their metabolic benefits will form the focus of the rest of this review. Importantly, neuronal activity patterns or expression of markers of active intracellular signaling are of limited use to map brain sites directly exposed to incretins, peripherally administered incretins, or incretin-based pharmacotherapies. First, GLP-1 is produced centrally and there is ample evidence that neurons producing GLP-1 are engaged in response to a peripheral bolus of GLP-1 or GLP-1R agonist (42). Second, intracellular signaling cascades downstream of incretin receptors recruit kinases and mediators shared with many other G protein-coupled receptors. Thus, we will focus on studies using labeled molecules to map brain access and central distribution of incretins and incretin-receptor agonists following peripheral administration.

### Can Incretin-Based Pharmacotherapies Cross the BBB?

A limited number of studies have quantified brain entry pharmacokinetics of GLP-1, GIP, or incretin-receptor agonists using brain perfusion of radiolabeled ligands and multiple time regression analysis, the gold standard technique to assess BBB permeability (55). Two groups employed this technique to characterize brain entry of GLP-1 and Exendin-4, a GLP-1R agonist, across the BBB and obtained converging conclusions. Brain entry of intravenously infused radiolabeled [Ser8]GLP-1, a stable analog of GLP-1, is not inhibited by excess doses of unlabeled [Ser8]GLP-1 or by the GLP-1 receptor antagonist exendin (9–39) in mice (56). Consistently,^125^I-labeled exendin-4 rapidly enters the brain in a nonsaturable manner at doses below 30 nmol/kg (57, 58). This suggests that GLP-1 and Exendin-4 can access the brain parenchyma through a nonsaturable, receptor-independent system, most likely via adsorptive transcytosis across brain endothelial cells. The lack of active receptor-mediated transport of GLP-1 across the BBB is also suggested by recent transcriptomic studies showing the lack of GLP-1R expression on hypothalamic endothelial cells or cells of the neurovascular unit (59) and the failure to detect GLP-1R immunoreactivity on brain endothelial cells using a highly-selective GLP-1R antibody with confocal or electron microscopy (60, 61). These results conflict with a study measuring active uptake of GLP-1 and Exendin 4 by rat brain immortalized endothelial cells through a receptor-mediated mechanism (62). Although this in vitro model is of lower physiological relevance, it leaves this question unclear. Using intravenous administrations of fluorescently labeled GLP-1 and Exendin 4, Fu et al. also observed reduced fluorescent signal intensity across different brain regions following pretreatment with a GLP-1R antagonist (62). Further validations would be needed to fully appreciate the sensitivity of this modified perfusion assay, which might be insufficient given that the expected interregional differences within the brain are not detected. Likewise,^125^I-labeled GLP-1 only labels the AP and subfornical organ (SFO, one of the CVOs) 5 min after transcardiac administration, and this labeling is abolished by an excess amount of unlabeled GLP-1, suggesting a receptor-dependent mechanism (63). Clearly in that timeframe, transport across the BBB is minimal, but this points toward the role of distinct mechanisms of brain access in acute conditions, which is discussed in more detail later in this review. Thus, GLP-1 and Exendin 4 can cross the BBB, most likely through slow and passive receptor-independent mechanisms.

Evidence for GIP transport across the BBB is even more scarce. We identified one study using radiolabeled GIP indicating that GIP crosses the BBB in a time-dependent saturable manner, inhibited by competition with unlabeled native GIP (64). This suggests that GIP employs a receptor-mediated transport mechanism to cross the BBB and access GIPR distributed throughout the brain (65). Although GIPR is expressed weakly in hypothalamic endothelial cells, it is highly enriched in pericytes (59), which might mediate transport function across the BBB (66). Bearing in mind the consequences of leptin signaling in pericytes on paracellular BBB transport (6), further work is needed to determine whether GIP signaling in brain pericytes also modifies BBB properties, perhaps contributing to the mechanism of action of incretin-based pharmacotherapies leveraging a GIP signaling arm. Of note, *Gip* transcript has been detected in discrete brain areas (67), but additional evidence is needed to determine whether GIP is produced in the brain, ideally using GIP-specific antibodies or a GIP reporter transgenic mouse. If these fail to confirm brain GIP expression, widespread GIPR expression might be used as circumstantial evidence to further indicate GIP transport across the BBB. A recent study using intravenously administered fluorescently labeled GIP reports very limited labeling outside the CVOs, speaking against quantitatively significant GIP transport across the BBB (68). However, using this technique to map brain distribution of hormones with well-established transport routes across the BBB such as leptin or insulin also results in limited parenchymal labeling and a signal almost exclusive to CVOs (12, 69). Therefore, the use of light-sheet microscopy for imaging fluorescently labeled peptides may not be ideal for drawing conclusions about blood–brain barrier (BBB) transport. This could be due to the perfusion and harsh chemical steps required for the histological processing of brain samples, which may result in the washing away of labeled compounds. In addition, the low resolution of image acquisition with the early generations of light-sheet microscopes, particularly when imaging large samples, presents another limitation. However, the latest technical innovations in this field are already enhancing this aspect and are expected to provide new insights in the near future.

The chemical modifications conferring resistance to degradation by DPP-IV and increasing drug half-life may alter the ability of incretin receptor therapies to access the brain (70). Using the brain perfusion technique and radiolabeled liraglutide, semaglutide, and other fatty acid acylated or PEGylated analogs of incretin-receptor agonists, Salameh et al. concluded that acylation and PEGylation prevent these molecules from crossing the BBB endothelium (58). This is consistent with a study using ^125^I-liraglutide to assess its entry through the BBB (51). This result is guiding the development of new drugs that leverage the beneficial effects of incretin-receptor activation on neurodegeneration (71), a topic outside the scope of this review. In the context of appetite regulation, acylation clearly enhances the therapeutic benefits of GLP-1R agonism (72), suggesting that the ability to cross the BBB is not critical for GLP-1R agonists to access functionally relevant sites for their anti-obesity action. Studies relying on the detection of peripherally injected fluorescently labeled GLP-1R agonists using light sheet microscopy in cleared brains provide a distinct picture on the effect of acylation on brain entry of GLP-1R agonists, likely BBB independent and therefore discussed later in this review.

### Can Incretin-Based Pharmacotherapies Cross the BCB?

Available evidence suggests that GLP-1R agonists do not cross the rodent BCB. Although most fluorescently labeled GLP-1R agonists label the choroid plexus, as all other CVOs (54, 60, 61, 69, 73), high-resolution microscopy indicates that labeling is absent from ependymal cells of the choroid plexus or lining the walls of the cerebral ventricles (74). In addition, choroid plexus labeling with intravenously administered fluorescently labeled GLP-1R agonists is maintained in GLP-1R knockout mice, indicating a receptor-independent signal (54). Consistent with the lack of BCB transport, labeled GLP-1 does not enter the rat CSF (62). Species differences might exist between rodents and humans, since both GLP-1R RNA and protein have been reported in human choroid plexus ependymal cells (75); however, only minimal quantities of liraglutide are detectable in the CSF of human participants treated for 5 mo or more with liraglutide, suggesting minimal access via the BCB (76).

Fluorescently labeled GIP also labels the choroid plexus (68), but additional work is needed to determine if GIPR is expressed on choroid plexus ependymal cells, and whether GIP can access the CSF through the BCB barrier.

### Brain Access of Incretin Receptor Agonists via CVOs

There is little ambiguity regarding the fact that incretin receptor agonists do access CVOs following peripheral administration (54, 68), but whether local sensing within CVOs is required or sufficient for their metabolic benefits is not so clear. The ME and AP are some of the brain sites with highest GLP-1R immunoreactivity (52) and the AP is also highly enriched in GIPR-expressing cells (65). Thus, incretin-receptor agonists might not even need to cross any brain barriers to produce their feeding and weight-suppressive effects. Robust cFos expression is observed in most GLP-1R and GIPR-expressing neurons following peripheral injections with their respective agonists (47, 65), indicating that local direct sensing is engaged, and there is good evidence indicating that AP GLP-1R signaling is sufficient and necessary to produce taste avoidance in response to GLP-1R agonism (41). In addition, AP GIPR signaling modulates this response (77, 78), suggesting that AP incretin receptor signaling is critical to balance the activity of downstream avoidance circuits, a mechanism potentially contributing to the enhanced efficacy of GLP-1R/GIPR co-agonism. However, AP lesions in rodents do not decrease the short-term anorectic or long-term weight loss effects of GLP-1R agonists (79, 80), and this is also true in mice with AP-specific ablation of GLP-1R-expressing neurons but are otherwise neuroanatomically intact (41). These data argue against a role for direct AP neuronal sensing in the metabolic benefits of GLP-1R agonists.

Functional consequences on ME incretin receptor signaling are not as straightforward to examine, since most ME cells are nonneuronal, and Gipr- or Glp1r-expressing cell bodies are mostly absent from this region (14, 65, 81). However, dense GLP-1R immunoreactivity can be detected in the ME (69). Recent ultrastructural examination of ME GLP-1R immunoreactivity using electron microscopy revealed the presence of GLP-1R on dendrites and on the surface of axon varicosities and axon terminals (52). Intriguingly, GLP1-R immunoreactivity was also localized to dense core transport vesicles in ME axons, suggesting potential trafficking of GLP-1R and internalization of GLP-1R/GLP-1R agonist complexes within axons (52). Whether axonal retrograde vesicular transport of such complexes (82), all the way to distal cell bodies, might form an access route for peripherally administered incretin receptor agonists is unknown. Furthermore, the presence of GLP-1R on terminals of hypophysiotropic axons of the ME suggests that peripheral GLP-1 or GLP-1R agonists might regulate the release of hypophysiotropic hormones into the portal capillaries and therefore the activity of neuroendocrine axes. In fact, GLP-1 administration rapidly leads to increased circulating levels of AVP and corticosterone in rats (83), but whether this neuroendocrine mechanism contributes to the metabolic benefits of GLP-1 or incretin-based weight loss drugs has not been confirmed. ME GIP signaling might also regulate the activity of neuroendocrine terminals, since dense YFP immunoreactivity is observed in the external zone of the median eminence in *Gipr-Cre:Rosa26-eYfp* reporter mice (65), but the presence of GIPR on these terminals needs to be confirmed. Likewise, incretin receptor signaling on neuronal processes projecting to the AP should be considered, since multiple neuronal populations from the NTS, dorsomotor nucleus of the vagus, and hypoglossal nucleus directly access the AP environment (84). Importantly, studies relying on AP lesions or AP-specific receptor knockdown do not address incretin receptor signaling on axons of dendrites of neurons residing outside the AP, highlighting the need for alternative approaches.

In addition to accessing the CVO environment through dendritic and axonal projections, neurons positioned in the vicinity of CVOs might be exposed to peripherally dosed incretin receptor agonists via diffusion through the CVO-parenchymal barriers. Fluorescently labeled GLP-1R agonists clearly diffuse from CVOs to adjacent parenchymal sites following peripheral injections (54, 61), and several studies support the relevance of direct ARH and NTS GLP-1R signaling in the acute and long-term appetite- and weight-suppressive effects of GLP-1R agonism (54, 80). In contrast, fluorescently labeled GIPR agonists remain confined to the ME and AP and do not label cell bodies in nearby nuclei (68), suggesting that GIPR-expressing neurons might not benefit from access through GIPR agonist diffusion across these barriers. However, this technique is not designed to measure BBB transport, its sensitivity depends on receptor expression levels (54), and low expression levels of GIPR in NTS and ARH neurons might account for the lack of labeling with peripherally dosed fluorescently labeled GIPR agonists.

Light-sheet imaging following the peripheral administration of fluorescently labeled incretin receptor agonists has been insightful in revealing the effect of acylation on brain entry of GLP-1R agonists through CVO-parenchymal diffusion barriers. Although labeled Exendin4 localizes exclusively to the CVOs, ARH, and NTS, various acylated versions of Exendin 4 are detected in hypothalamic nuclei more distal from the ME, as well as deep parenchymal regions such as the medial Habenula, lateral septal nucleus, or dentate gyrus, with highest penetration observed for C18-diacid Exendin-4 (60). Likewise, semaglutide, also C18-diacetylated, shows increased brain penetration compared to liraglutide (61). Mechanistically, how acylation might increase brain entry warrants further evaluation, but might involve differential receptor internalization and intracellular trafficking (85). By increasing lipophilicity and albumin-binding affinity, acylation might enhance passive diffusion across the BBB or adsorptive transcytosis (60), but this would not be consistent with studies showing lack of transport across the BBB of radiolabeled acylated agonists (58). Instead, diffusion across barriers between CVOs and adjacent parenchymal regions might be enhanced. In favor of this mechanism, all studies mapping the central distribution of fluorescently labeled GLP-1R agonists report that peripherally administered compounds clearly label CVOs with the highest intensity and diffuse from these sites in a gradual manner with more or less efficiency (60, 61, 73). Thus, different diffusion properties across CVO-parenchymal barriers rather than enhanced BBB permeability might account for the effect of acylation on brain access of GLP-1R agonists. For example, diffusion across the ME-ARH barrier and within the ARH is strikingly improved with C18-dialcetylation (60, 61). However, additional compound-selective mechanisms are likely recruited, accounting for the access of GLP-1R agonists to sites distal from CVOs like the paraventricular nucleus of the hypothalamus (PVH) or supraoptic nucleus (73), and perhaps explaining the enhanced diffusion of liraglutide versus semaglutide within the PVH (61, 73), while exendin-4 does not access the PVH (54, 74). With regards to the effect of acylation of the diffusion of GIPR agonist, to our knowledge, this has not been investigated to date.

Recent evidence suggests that peripherally administered GLP-1R agonists enter the brain via transcytosis across β2-tanycytes, a subpopulation of ependymal cells lining the floor of the third ventricle and forming the ME-CSF barrier (11). This is an attractive hypothesis to account for enhanced exposure of periventricular GLP-1R-expressing neuronal populations to GLP-1R agonists, and this mechanism has also been proposed to mediate the entry of other blood-borne metabolic hormones into the brain (86). Fluorescently labeled liraglutide rapidly labels cell bodies of β2-tanycytes following intravenous injection (51). In addition, in mice with either inducible expression of botulinum toxin in tanycytes via ventricular TAT-Cre injections to impair tanycytic vesicular exocytosis, or *Glp1-r* knockdown using ventricular shRNA injections, the acute anorectic response to liraglutide is blunted (51). However, two lines of arguments speak against a role for β2-tanycytes in the mechanisms of brain access of GLP-1R agonists to their brain targets for weight loss. First, immunodetection of GLP-1R at high subcellular resolution using electron microscopy indicates no or sparse GLP-1R-immunoreactivity in β2 tanycytes (52, 61). In addition, GLP-1R-dependent internalization is not necessary for the diffusion of GLP-1R agonists to the ARH or NTS, since labeled Exendin-9 has the same brain distribution as labeled exendin-4 (54, 60, 74). Intriguingly, although liraglutide-induced cFos activation in the ARH is unaffected in models where GLP-1R expression in β2-tanycytes is blunted, it is significantly reduced in the PVH and LH in these conditions, suggesting that β2-tanycytic transport might be relevant for access to sites more distal from the ME. Thus, further work is needed to fully establish the functional relevance of β2-tanycytes in brain access of blood-borne GLP1-R agonists and characterize the underpinning mechanisms.

## CONCLUSIONS

Incretin-based therapies create unprecedented opportunities to treat obesity and associated cardiometabolic disorders with high efficiency and minimal side effects, motivating new research efforts to increase our understanding of the central mechanisms of action of these drugs and the routes through which they access their functional brain targets to produce sustained weight loss. Recent findings have highlighted the role of the area postrema as the neuroanatomical substrate through which incretin receptor agonism regulates avoidance responses, but the neural mechanism through which they mediate sustained weight loss remains unclear. Available evidence indicates that efficient weight loss does not require transport across the BBB. Instead, specialized access mechanisms from the CVOs are recruited, allowing exposure of adjacent as well as more distal brain sites. The ability of various GLP-1R agonists to access parenchymal areas is heterogeneous, pointing to a restricted list of brain sites which should be further evaluated for their functional contribution to the weight loss response. Similar studies assessing brain access through the CVO routes by GLP-1R/GIPR co-agonists might help further refine functionally relevant access mechanisms and target sites. The possibility that GIPR agonism might enhance the weight-suppressive action of GLP-1R agonists by modifying brain access to functionally relevant cells should be further evaluated. The existing literature crucially lacks studies examining transport mechanisms in mice with obesity and characterizing the consequences of positive energy balance on relevant access mechanisms. Hints that access of Exendin-4 is altered in high-fat high-sucrose fed mice highlights the need for more studies performed under these conditions (69). In addition, mechanisms and efficiency of brain access might change during the course of chronic treatments since plasma membrane expression of incretin receptors is known to decrease following sustained exposure to incretin receptor agonists. The metabolic benefits associated with chronic treatment might also contribute to changes in brain access over time. For example, decreased glycemia has been proposed to increase diffusion of Exendin-4 to the ARH and NTS (69). Thus, the investigation of brain access of incretin-based weight loss compounds should create new opportunities to increase our mechanistic understanding and novel rationales guiding the design of the next generation of this class of weight loss agents.

## GRANTS

This work was supported by a Medical Research Council grant (MT/S011552/1) and a Diabetes UK grant (22/0006401).

## DISCLOSURES

No conflicts of interest, financial or otherwise, are declared by the authors

## AUTHOR CONTRIBUTIONS

S.B. and C.B. drafted manuscript; S.B. and C.B. edited and revised manuscript; S.B. and C.B. approved final version of manuscript.
